# Pose Estimation and Behavior Classification of Jinling White Duck Based on Improved HRNet

**DOI:** 10.3390/ani13182878

**Published:** 2023-09-10

**Authors:** Shida Zhao, Zongchun Bai, Lili Meng, Guofeng Han, Enze Duan

**Affiliations:** 1 Institute of Agricultural Facilities and Equipment, Jiangsu Academy of Agricultural Sciences, Nanjing 210014, China; 2 Key Laboratory of Protected Agriculture Engineering in the Middle and Lower Reaches of Yangtze River, Ministry of Agriculture and Rural Affairs, Nanjing 210014, China; 3 School of Civil Engineering, Engineering Campus, Universiti Sains Malaysia, Nibong Tebal 14300, Malaysia

**Keywords:** breeder duck, computer vision, pose estimation, keypoints, HRNet-32 network, attention mechanism, poultry breeding

## Abstract

**Simple Summary:**

The behavior and pose of ducks during the breeding process are directly related to their welfare and health status. The real-time and accurate monitoring of their different behaviors and poses is significant. In this study, we proposed a duck pose estimation method using computer vision techniques, achieving accurate detection of eight keypoints, including the head, beak, chest, left leg, right leg, left foot, right foot, and tail, under six behaviors: standing, drinking, preening, spreading wings, walking, and resting, and forming correct body expressions. Furthermore, the model’s generalization capability was tested with different lighting intensity levels. Ducks of various ages, breeds, and farming methods were used to validate the model’s comprehensive pose estimation capability. The model’s comprehensive detection capability was compared with mainstream pose estimation methods, proving its superiority. The results of this study are considered instrumental in smart poultry farming.

**Abstract:**

In breeding ducks, obtaining the pose information is vital for perceiving their physiological health, ensuring welfare in breeding, and monitoring environmental comfort. This paper proposes a pose estimation method by combining HRNet and CBAM to achieve automatic and accurate detection of duck’s multi-poses. Through comparison, HRNet-32 is identified as the optimal option for duck pose estimation. Based on this, multiple CBAM modules are densely embedded into the HRNet-32 network to obtain the pose estimation model based on HRNet-32-CBAM, realizing accurate detection and correlation of eight keypoints across six different behaviors. Furthermore, the model’s generalization ability is tested under different illumination conditions, and the model’s comprehensive detection abilities are evaluated on Cherry Valley ducklings of 12 and 24 days of age. Moreover, this model is compared with mainstream pose estimation methods to reveal its advantages and disadvantages, and its real-time performance is tested using images of 256 × 256, 512 × 512, and 728 × 728 pixel sizes. The experimental results indicate that for the duck pose estimation dataset, the proposed method achieves an average precision (AP) of 0.943, which has a strong generalization ability and can achieve real-time estimation of the duck’s multi-poses under different ages, breeds, and farming modes. This study can provide a technical reference and a basis for the intelligent farming of poultry animals.

## 1. Introduction

Healthy breeding of breeding ducks improves egg production rate, hatchability, and meat quality, enhances the economic efficiency and market competitiveness of duck meat and eggs, and reduces farming losses. However, the duck breeding process faces such problems as high breeding costs, low efficiency, and significant losses. Scale and intelligence have become inevitable trends in the development of the duck breeding industry [1,2]. However, in the context of gradually increasing farming density, the probability of abnormal conditions and disease in ducks also increases. Therefore, timely, efficient, and accurate perception of abnormalities and disease prevention are key to scaled farming. Studies have shown a significant relationship between animal behavioral changes and their internal and external physiological health status. By measuring and analyzing animal behavior data, it is possible to accurately reflect their physiological health changes, which is an important step toward the scientific and rigorous development of animal behavior [3,4,5]. Building upon this, researching the identification and analysis of duck behavior can provide new ideas and methods for detecting its abnormalities and diseases. Since duck behavior consists of different combinations of poses, achieving accurate pose estimation is the basis and prerequisite for their body information extraction and movement analysis. Based on this foundation, researching duck pose estimation methods is significant for breeder duck breeding monitoring and perceiving health.

When conducting behavior or pose detection in poultry using traditional methods, it is common to utilize accelerometers [6,7,8], RFID [9,10,11], or other physical markers attached to various parts of the poultry, such as their back or legs, to capture their motion information. However, this approach is costly, cumbersome, and easily causes rejection, which is not conducive to improving poultry’s physiological health and animal welfare. In addition, with the change in poultry size and long-term movements, it is easy to lead to sensor loss, with poor practicability and high limitations [12]. In recent years, with the rapid development of deep learning technology, computer vision algorithms based on deep learning have demonstrated powerful learning and inference capabilities in object recognition and detection. This makes it possible to explore the use of this method to solve the above problems.

Currently, many scholars research behavior recognition [13,14] and individual detection [15,16,17,18] in poultry using computer vision techniques. Guo et al. [19] proposed an automatic and accurate recognition method for four behaviors of feeding, drinking, standing, and resting in broilers of different breeding ages in chicken houses by introducing different CNN networks for comparative analysis. Gu et al. [20] proposed a duck neck extension, trampling, and wing spreading behavior recognition model based on YoloV5 to assess caged egg-laying ducks’ health and welfare status. Xiao et al. [21] introduced a monitoring method for the efficient and accurate detection of key behaviors, such as feeding and drinking, in group-raised Magang geese using YOLO-X. Zheng et al. [22] proposed an algorithm for measuring chickens’ calf length and girth to improve the efficiency of measuring chicken phenotypic parameters. Although the above studies can achieve typical behavior recognition and detection of key parts in poultry, achieving duck pose estimation involves behavior and keypoint detection and requires considering the correlation of various parts of the duck’s body. Therefore, the above-mentioned technical achievements cannot meet the requirements for duck pose estimation.

Convolutional neural network-based pose estimation algorithms have been widely applied in detecting key parts and action recognition of animals [23,24,25,26]. Fang et al. [27] achieved the accurate recognition of multiple poses of broiler chickens using deep convolutional neural networks (DNNs). Zhang et al. [28] introduced a transformer-based animal keypoint detection model for sheep. Xue et al. [29] proposed a 2D+3D-CNet by fusing the 2D–3D convolution features of the image to realize the accurate recognition of the pose change in lactating sows. Mathis et al. [30] proposed a novel pose estimation method, DeepLabCut, by a deep neural network, achieving unsupervised real-time detection of various poses in multiple animals, such as horses, dogs, and lions. The above studies have demonstrated the excellent applicability of computer vision techniques in animal pose estimation. However, comprehensive results on duck pose estimation technology applications are scarce. With the popularization of the scaled cage-reared mode for ducks and the frequent occurrence of abnormal duck behavior, there is an urgent need for innovative approaches to duck pose estimation methods, in order to provide a technical foundation for future intelligent duck breeding.

In summary, this paper addresses the practical needs in the breeding process of breeding ducks using HRNet as the base network and focuses on the following four areas of research: (i) proposing a breeding duck pose estimation model with high accuracy, multiple keypoints, and multiple behaviors; (ii) exploring the embedding of CBAM in the network structure to optimize the detection accuracy of the model; (iii) testing the generalization ability of the model and its pose estimation performance for ducks of different reproductive ages; and (iv) comparing and analyzing the advantages and disadvantages of the proposed model by introducing other mainstream pose estimation algorithms, and testing the real-time performance of the model. The proposed method of duck pose estimation can not only provide technical support for breeder duck welfare breeding and abnormality monitoring but also serve as a technical reference for the smart breeding of other poultry species.

## 2. Materials and Methods

### 2.1. Experimental Materials and Image Acquisition

This paper selected the research subject of the Jinling white duck, aged between 6 and 8 weeks, free from any diseases, and meeting quarantine requirements. The Jinling white duck is a high-quality native breed based on the Nanjing Animal Husbandry and Poultry Science Research Institute, which is a crossbreeding and improvement of the Liancheng white duck and the Cherry Valley duck and is characterized by a fast growth rate and large carcass weight [31,32]. Video samples of the breeding process of the Jinling white duck were collected from the Jiangning base of the Nanjing Animal Husbandry and Poultry Science Research Institute, where the breeding mode is ground-level breeding, and the height of the duck house surrounding wall is 1.1 m. RealSense D435i cameras were set up at 0.35 m from the wall and 1.6 m from the ground around the duck shed. Additionally, the camera’s field of view and depth of field were adjusted by angling the gimbal downwards at 27° to capture as many duck postures as possible. During video acquisition, there was no specific light source or shooting time. To prevent stress damage to ducks caused by environmental mutation, cameras and brackets are arranged in advance to enable ducks to adapt, and personnel remotely control the video acquisition program.

**Figure 1:**
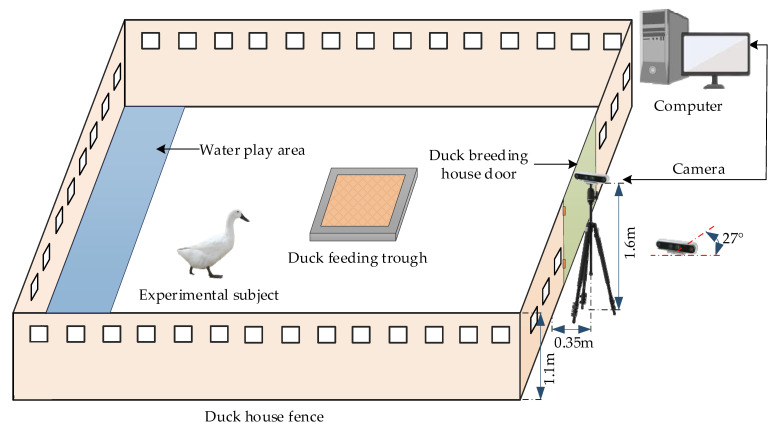
Layout of Jinling white duck poses video acquisition device.

In addition, to increase the diversity of duck samples and improve the generalization ability of the later model, this paper performed video acquisition in 3 duck pens, each containing 182, 378, and 220 ducks. Subsequently, manual frame extraction was conducted every 5–6 frames, and the images were cropped to include only one duck per frame. After selection, a total of 3800 images of Jinling white duck were obtained, with a size of 1920 × 1080 pixels. The layout of the Jinling white duck pose video acquisition is shown in Figure 1.

### 2.2. Annotation of Jinling White Duck Images and Establishment of the Dataset

**Figure 2:**
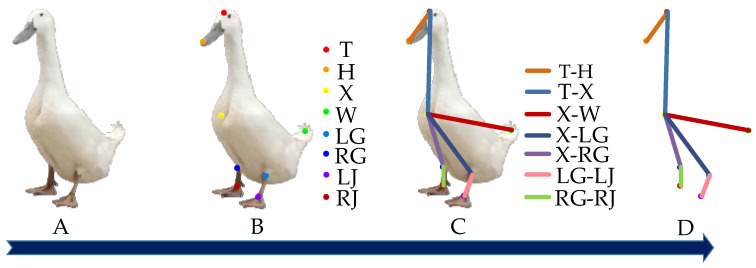
Example of Jinling white duck keypoints annotation. Note: (**A**) represents the duck image, (**B**) represents the locations of the keypoints in the duck image, (**C**) depicts the distribution of the keypoint connections in the duck image, and (**D**) represents the duck’s body represented by the keypoints and connections.

The key parts of the duck, such as the head, beak, chest, legs, and feet, are connected in an orderly manner as a skeleton, which can completely characterize the duck’s body. This paper used the Labelme image annotation tool to annotate the objects of the Jinling white duck in the images. A total of eight keypoints were annotated, including the head, beak, chest, left leg, right leg, left foot, right foot, and tail, denoted by different colors as T, H, X, LG, RG, LJ, RJ, and W, respectively. After that, the head is connected to the beak (T-H), the head is connected to the chest (T-X), the tail is connected to the chest (W-X), the left leg is connected to the left foot (LG-LJ), the right leg is connected to the right foot (RG-RJ), and the keypoints are associated to form the duck body. The description and definition of the keypoints of the duck, as well as the labeling examples, are presented in Table 1 and Figure 2, respectively.

**Figure 3:**
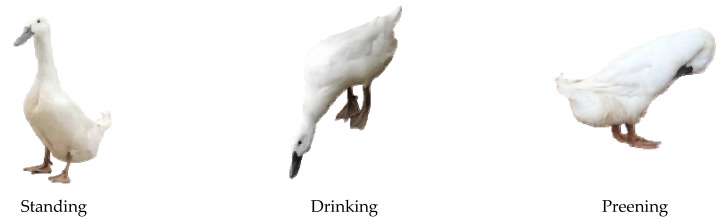

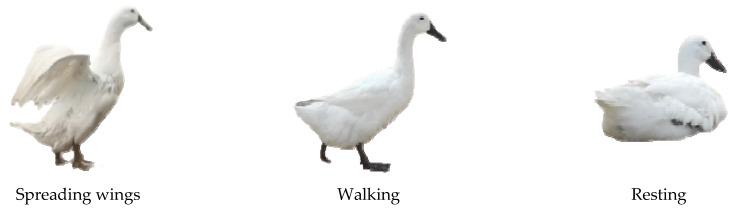
Example of six types of poses in the Jinling white duck pose estimation dataset.

After the keypoints of the acquired duck images are annotated, the training set and test set are divided in a 7:3 ratio to construct the pose estimation dataset. Due to the diverse natural physiological poses of ducks, the pose judgment standard was established by experienced duck breeders, including standing, drinking water, thinning feathers, spreading wings, pacing, and lying down, with a total of 6 poses. After manual statistics, the proportion of images in the pose mentioned above in the dataset approaches 5:2:4:1:6:1. The description and examples of duck poses are presented in Table 2 and Figure 3.

### 2.3. Pose Estimation Method for Jinling White Duck

The University of Science and Technology of China (USTC) and Microsoft Research Asia proposed the high-resolution network (HRNet) in 2019 [33]. This network can maintain high-resolution feature maps throughout the feature extraction process, resulting in outstanding performance in pose estimation [34,35] and object detection [36,37]. Furthermore, based on the accuracy advantage of the top-down strategy-based pose estimation method [38], as well as the impact of introducing attention mechanisms on improving the network accuracy, this paper uses HRNet as the base network. Following the top-down strategy, it introduces attention mechanisms to conduct pose estimation research on the Jinling white duck.

#### 2.3.1. Pose Estimation Network for Jinling White Duck Based on the Improved HRNet

**Figure 4:**
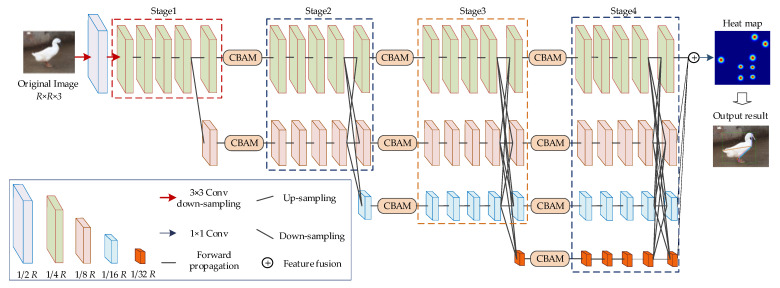
Structure of Jinling white duck pose estimation network based on improved HRNnet.

The original HRNet network consists of four stages, which include different resolutions, parallel connections, and multiscale feature concatenation, aiming to achieve feature parsing and localization. However, the multiple fusion of features at different resolutions may introduce noise, cause information redundancy, and increase the computational complexity of the network. This paper introduces the convolutional block attention module (CBAM) [39] to each stage’s feature output parts to address these issues, enhancing contextual information. This approach maximizes the preservation of keypoint information of ducks while improving the network’s capability to extract crucial details from the fused features. The structure of the proposed Jinling white duck pose estimation network in this paper is shown in Figure 4.

As shown in Figure 4, the proposed network starts from Stage 2, and each stage sequentially adds a parallel branch, following the rule that the output of the nth branch serves as the input for the (n + 1)th branch. Additionally, the number of channels in the new branches doubles, and the resolution is half the lowest resolution branch in the previous stage. Therefore, the feature map resolutions for Stage 1 to Stage 4 are 1/4, 1/8, 1/16, and 1/32 of the original image.

Moreover, dense upsampling and downsampling operations are performed for cross-fusion of features between the output of each stage branch and the input of the next stage. A convolution with a kernel size of 3×3 stride 2 and ReLU activation is used for upsampling, implementing the convolution operation. For downsampling, convolution with a kernel size of 3×3 stride 1 and ReLU activation is employed, aligning with the higher resolution feature tensor from the previous stage using bilinear interpolation. After the feature fusion in Stage 1 to Stage 3, a total of 9 serial CBAM modules are embedded into each branch, enabling feature recalibration in both the channel and spatial dimensions. After that, the output features of each branch in Stage 4 are upsampled, followed by ReLU activation and convolution with a kernel size of 3×3 and stride 1, resulting in feature maps with a resolution of 1/4 R. Because this experiment involves a total of 8 types of duck keypoints, the depth of the feature map is set as 8. Finally, the keypoint positions are obtained, and based on the defined connection rules, the final Jinling white duck keypoints and body information are outputted into the original image.

#### 2.3.2. CBAM (Convolutional Block Attention Module) Attention Mechanism

**Figure 5:**
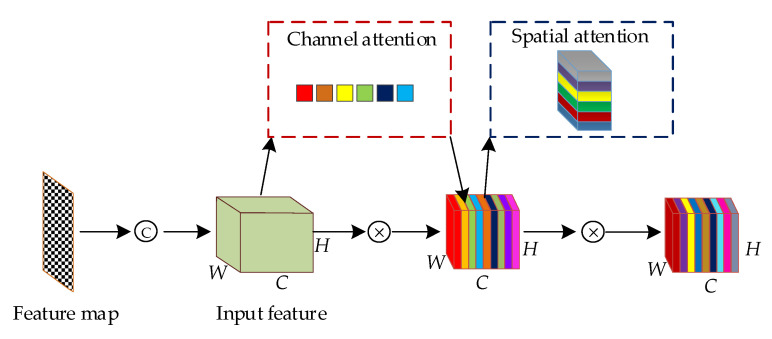
General structure diagram of CBAM. Note: Ⓒ is a convolution, ⮾ represents multiply.

The original HRNet performs feature fusion on different resolution branches through dense upsampling and downsampling, aiming to preserve the diversity and richness of coarse-grained and fine-grained features in ducks. However, it neglects the importance of different network channels. Meanwhile, in this experiment, the duck’s multi-class keypoints need to be accurately detected, localized, and formed into correct logical associations, which puts high demands on the overall and local detailed feature extraction of the duck’s body. The CBAM’s channel attention and spatial attention composition, cascade structure, global average pooling operation, and attention weight adaptation make it able to meet the research requirements of this experiment. Therefore, this experiment enhances the feature extraction capability of HRNet by embedding CBAM to improve the detection accuracy of the later model. CBAM consists of two concatenated parts: the channel attention module (CAM) and the spatial attention module (SAM). The overall structure of CBAM is shown in Figure 5. Since CBAM is a lightweight and general-purpose module, the additional computational cost it introduces is negligible.

**Figure 6:**
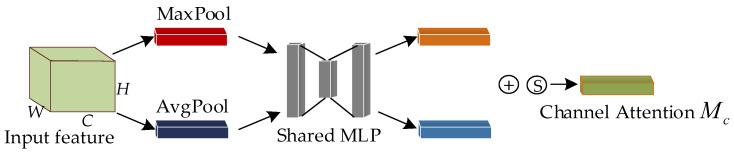
Channel attention structure of CBAM. Note: ⊕ represents the summation of elements, Ⓢ represents the Sigmoid function.

**Figure 7:**
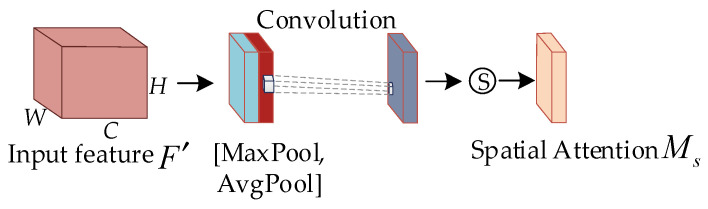
Spatial attention structure of CBAM.

After the convolution operation, the feature map of the Jinling white duck is expanded in channel size, resulting in an input feature of size W×C×H. The input feature undergoes the channel attention mechanism to obtain channel attention weights. Simultaneously, it is elementwise multiplied with the channel attention weights and then interacts similarly with the spatial attention mechanism. Finally, the spatial attention weights are multiplied with the feature map that has undergone channel attention adjustment, resulting in the refined feature through adaptive feature extraction. The calculation steps are shown in Equations (1) and (2), and the structures of channel attention and spatial attention are illustrated in Figure 6 and Figure 7, respectively.

(1) $$F^{\prime }=M_{c}(F)⊗F$$

(2) $$F^{\prime \prime }=M_{s}(F)⊗F^{\prime }$$

In the equation, F represents the input feature, ⊗ denotes element-wise multiplication, $F^{\prime }$ is the feature obtained by F weighted by the channel attention mechanism, $M_{c}$ represents the function for calculating channel attention, $F^{\prime \prime }$ denotes the feature obtained by weighting $F^{\prime }$ through the spatial attention mechanism, and $M_{s}$ represents the calculation function of the spatial attention mechanism.

The channel attention module first performs global maximum pooling and global average pooling on the width and height of the input features, compresses the feature maps into 1×1×C the format, and transfers the two compressed feature maps to a shared fully connected multilayer perceptron (*MLP*) for processing, after which the output results are summed. The sigmoid function is used to obtain the channel attention weights (0–1) and finally generates the channel attention features $F^{\prime }$.

Unlike the channel attention module, the spatial attention module performs average pooling and maximum pooling to each feature point of the feature $F^{\prime }$ and convolutions the results to adjust the number of feature channels to 1. After that, the sigmoid function is applied to obtain the weight values (0–1) for each feature point of the input feature map. Finally, the spatial attention weights are multiplied channelwise with the transmitted features to obtain $F^{\prime \prime }$. The computation processes for $M_{c}$ and $M_{s}$ in the diagram are shown in Equations (3) and (4), respectively.

(3) $$M_{c}(F)=\sigma (MLP(AvgPool(F))+MLP(MaxPool(F)))$$

(4) $$M_{s}(F)=\sigma (f^{n\times n}[AvgPool(F);MaxPool(F)]))$$

In the equation, σ represents the sigmoid function, MLP represents a multilayer perceptron, AvgPool represents average pooling, MaxPool represents maximum pooling, and $f^{n\times n}$ represents a convolution operation with a kernel size of n×n.

### 2.4. Image Brightness Adjustment Methods

To simulate duck images under different lighting conditions and analyze the generalization ability of duck pose estimation models, the OpenCV image processing function library was used to convert the RGB color space of duck images into HSV. Subsequently, the V channel value representing the image’s brightness is assigned to multiple factors of 1.35 and 0.75 to represent the “brightened” and “darkened” images, respectively.

(5) $$\left\{\begin{array}{c} lighten=V\times 1.35\\ darken=V\times 0.75 \end{array}\right.$$

### 2.5. Establishment of Different Breeding-Age Duck Image Datasets

**Figure 8:**
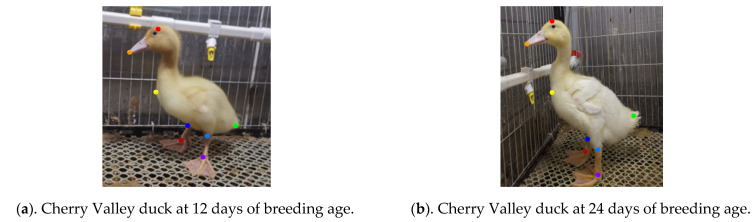
Examples of cherry valley ducks of different breeding ages.

To test the keypoints detection performance of the duck pose estimation model on different breeds of ducklings under other farming modes, the experiment took every 12 days as a time gradient and collected images of Cherry Valley meat ducks at 12 and 24 days of breeding age in cage farming mode. After manual preprocessing, a total of 300 duckling images were selected to establish duck image datasets for different breeding ages, breeds, and farming modes. The keypoint definition and skeleton association rules of the duckling are the same as those of the adult Jinling white duck, and the keypoint annotation process remains consistent. An example of duckling keypoints is shown in Figure 8a,b. There were significant differences in feather color and body size between ducklings at 12 and 24 days of age.

### 2.6. Performance Evaluation Criteria for Duck Pose Estimation

The commonly used AP (average precision) in pose estimation research is selected as the evaluation indicator for quantifying the detection accuracy of duck pose estimation models. The value of this indicator is between 0 and 1, and the larger the value is, the better the detection effect of the model [40]. Before calculating AP, it is necessary to determine the threshold value for object keypoint similarity (*OKS*), calculated according to Equation (6).

(6) $$OKS=\frac{\sum_{i}\exp(-\frac{d_{i}^{2}}{2s^{2}k_{i}^{2}})\delta (v_{i}>0)}{\sum_{i}\delta (v_{i}>0)}$$

In the formula, i is the keypoint number, $d_{i}$ is the Euclidean distance between the true value and predicted value of the keypoint with number i, $v_{i}$ is the visibility marker of the keypoint with number i, invisible is 0, occluded is 1, visible is 2, δ is the Kronecker delta function, $k_{i}$ is the constant of the keypoint with number i, and s is the object scale.

In the context of keypoint detection, a prediction is considered a true positive if its intersection over union (IoU) value exceeds a predefined threshold. Otherwise, it is considered a false positive. Based on this criterion, the AP of duck keypoint predictions can be computed, as shown in Equation (7).

(7) $$\mathrm{AP}=\frac{\sum_{p}\delta (OKS>S)}{\sum_{p}1}$$

In the equation, p represents the duck detection box index, and S denotes the threshold value. In this paper, the threshold value is set to 0.5.

To meet the practical needs of production, the duck pose estimation model should have high detection accuracy and demonstrate good real-time performance. Therefore, in this paper, the processing time of a single image is selected as an indicator to evaluate the real-time performance of the model. The calculation process can be summarized as follows: the average processing time for quantitative images is computed, and this process is repeated three times to obtain the final average. The unit is images per second, and a smaller value indicates better real-time performance, while a larger value indicates poorer real-time performance. Moreover, this study considers the memory size occupied by the model as an additional metric to evaluate its embeddability performance. The unit is in megabytes (MB), and a smaller memory value indicates a stronger embeddability of the model, while a larger memory value indicates a weaker embeddability.

### 2.7. Experimental Environment

A Lenovo brand custom computer was chosen as the experimental hardware setup. The CPU used was an AMD Ryzen 5 with a clock frequency of 3.90 GHz, and the system had 16 GB of RAM. An NVIDIA RTX3060 GPU with 12 GB of dedicated memory was also installed. Python was used to build a virtual environment based on the PyTorch deep learning framework and conduct model training and performance measurement.

### 2.8. Experimental Steps for Jinling White Duck Pose Estimation

The experimental procedure for Jinling white duck using the improved HRNet consists of six steps:

1. Data collection and annotation: Collecting and annotating Jinling white duck pose images and establishing a pose estimation dataset. Two variants of the HRNet network are used, and transfer learning is applied to train and compare the models on the dataset, resulting in the Jinling white duck pose estimation model.
2. Structural optimization: Embed multiple CBAM modules into the HRNet network at various positions for multiresolution feature fusion, optimizing the model’s structure.
3. Generalization ability test: Adjust the brightness of duck images to evaluate the model’s generalization ability.
4. Multi-scene detection ability test: Duck pose images were collected from different farming modes, breeds, and age groups to test the model’s comprehensive detection ability.
5. Comparative analysis: Compare the improved HRNet model with mainstream pose estimation methods to evaluate its advantages and disadvantages.
6. Real-time performance test: Test the model’s real-time performance by adjusting the image resolutions.

**Figure 9:**
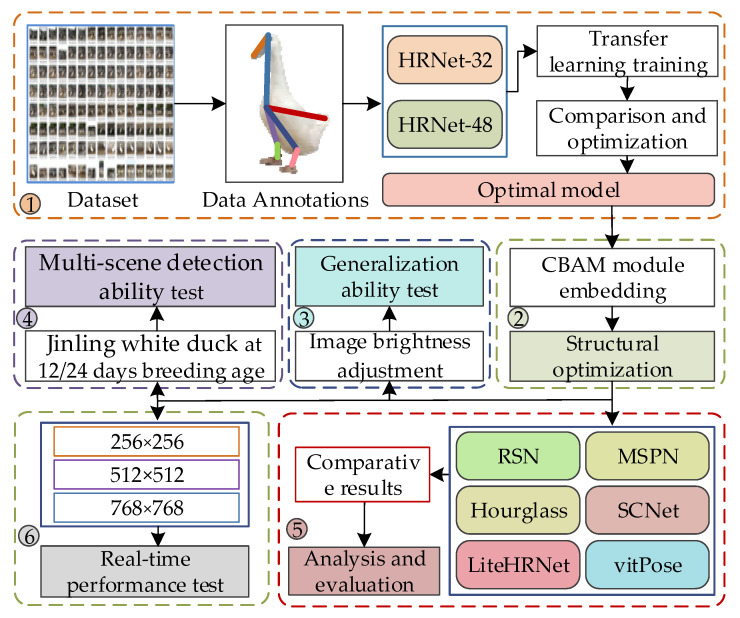
Experimental procedure of pose estimation and behavior classification for Jinling white duck based on improved HRNet.

The experimental procedure is illustrated in Figure 9.

## 3. Results

### 3.1. Jinling White Duck Pose Estimation Results Based on the Original HRNet

HRNet-32 and HRNet-48 were derived by changing the width of the high-resolution subnetworks of Stage 2–Stage 4 in the structure of HRNet. The width of Stage 2 to Stage 4 in HRNet-32 and HRNet-48 is as shown in Table 3. Due to changes in network structure and feature map size, there are differences in the performance of different HRNets in detecting duck keypoints. To obtain the optimal model, it is necessary to compare and analyze the performance of HRNet-32 and HRNet-48 in Jinling white duck pose estimation.

**Figure 10:**
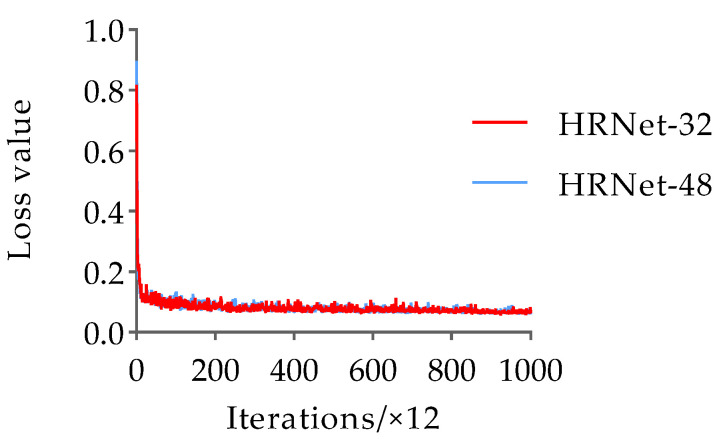
The trend in loss value variation with iteration between HRNet-32 and HRNet-48 during the training process.

**Figure 11:**
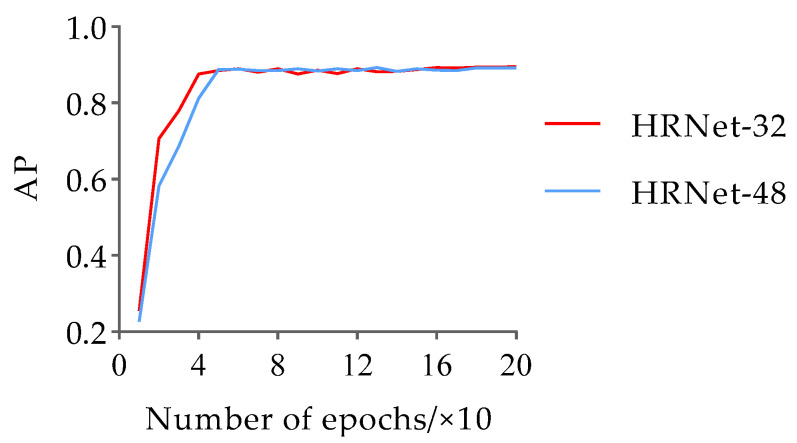
The trend in AP variation with iteration between HRNet-32 and HRNet-48 during the training process.

To prevent overfitting and convergence difficulties during the network training process, this paper loaded pre-training weights based on the animal pose dataset training and employed the adaptive moment estimation (Adam) optimizer with momentum and weight decay set to 0.9 and 0.0001, respectively. The learning rate and batch size were set to 0.001 and 16, respectively. Additionally, the model training followed the strategy of saving the model once per cycle, with a total of 12,000 iterations and 200 epochs. For the pose estimation dataset, the variation trends of the loss and AP values with the number of iterations during the training process of the HRNet-32 and HRNet-48 networks are shown in Figure 10 and Figure 11, respectively.

The results in Figure 10 and Figure 11 indicate that the trends of HRNet-32 and HRNet-48 loss values with the number of iterations are the same for the pose estimation dataset. These loss values rapidly decrease during the initial stages of training, followed by a slower decline starting at approximately 2400 iterations, eventually converging to approximately 0.063 in the form of slight oscillations. In addition, the AP values of HRNet-32 and HRNet-48 increased rapidly during the first four epochs, and the AP value of HRNet-32 was larger than that of HRNet-48. In the stage of 5–15 epochs, the AP values of the two fluctuated in the range of 0.100. Subsequently, after 16 epochs, their AP values become nearly identical and remain stable until the end of training. Finally, the AP values of HRNet-32 and HRNet-48 converged to 0.895 and 0.891, respectively. Based on the comprehensive results, the AP values of the duck pose estimation models obtained based on the two structural variants of HRNet are similar, and both are less than 0.900. Therefore, the detection performance needs to be further improved. However, considering the 0.004 higher AP value of HRNet-32 compared to HRNet-48, the experiment selects HRNet-32 as the base network for pose estimation of the Jinling white duck and conducts subsequent optimizations on its structure to enhance its accuracy.

### 3.2. Jinling White Duck Pose Estimation Results Based on the Improved HRNet

**Figure 12:**
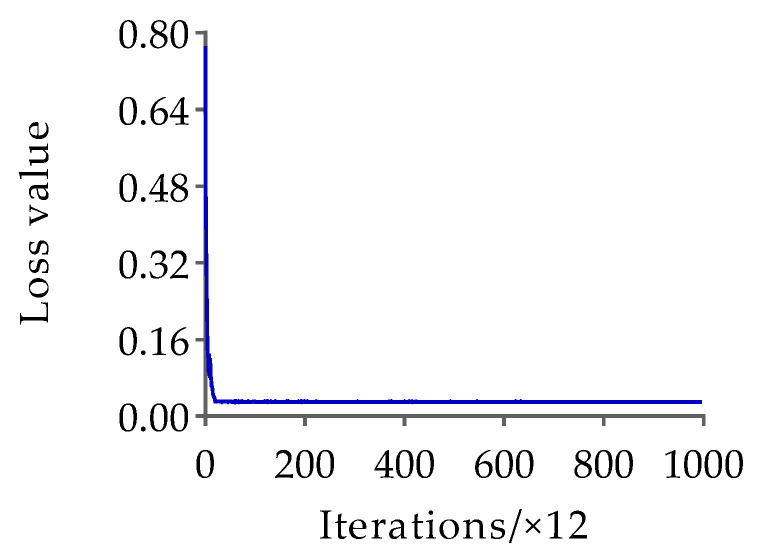
The variation of loss with iteration during the training process of the HRNet-32-CBAM.

**Figure 13:**
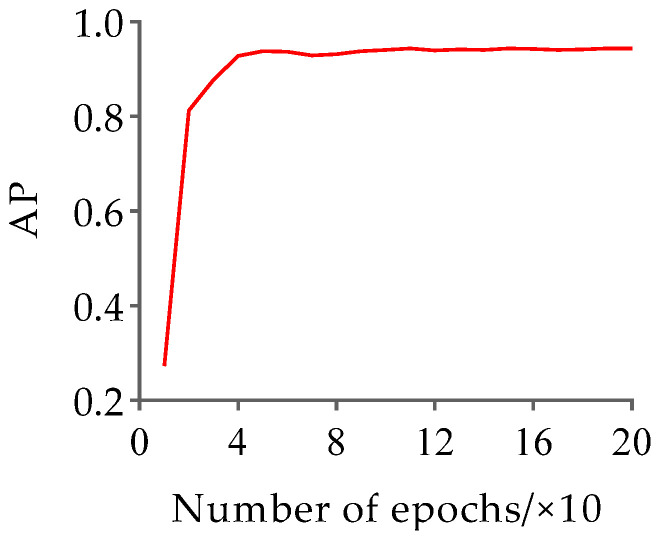
The variation of AP with iteration during the training process of the HRNet-32-CBAM.

Based on the HRNet-32-CBAM network proposed in Section 2.3.1, training was conducted on the pose estimation dataset to obtain the Jinling white duck pose estimation model. The hyperparameter settings are consistent with the original HRNet to ensure experimental consistency. The results of the changes in loss and AP values with the number of iterations during the training process of the HRNet-32-CBAM network are shown in Figure 12 and Figure 13.

**Figure 14:**
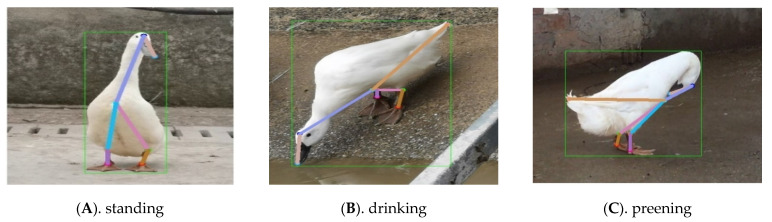

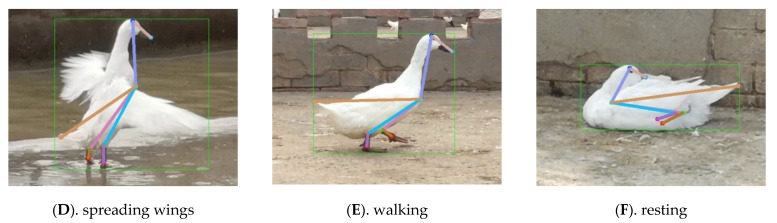
Pose estimation results of Jinling white duck based on HRNet-32-CBAM.

According to the results in Figure 12 and Figure 13, both the loss value and AP value show a significant decreasing and increasing trend in the early stages of training, and both gradually stabilize with increasing iteration times. Finally, the pose estimation model based on HRNet-32-CBAM achieved the highest AP value at the 16th epoch, reaching 0.943, which is a 0.048 improvement compared to HRNet-32. Therefore, this model was selected for pose estimation of the Jinling white duck and all subsequent performance tests. In addition, during the stage of extracting keypoint features and inferring poses of ducks, the experiment selects individual ducks with unobstructed and obvious poses as the input of the HRNet-32-CBAM network, in order to ensure that the model learns the different categories of the pose features of ducks, and to validate the model’s performance based on this. Some examples of Jinling white duck pose estimation results are shown in Figure 14.

**Figure 15:**
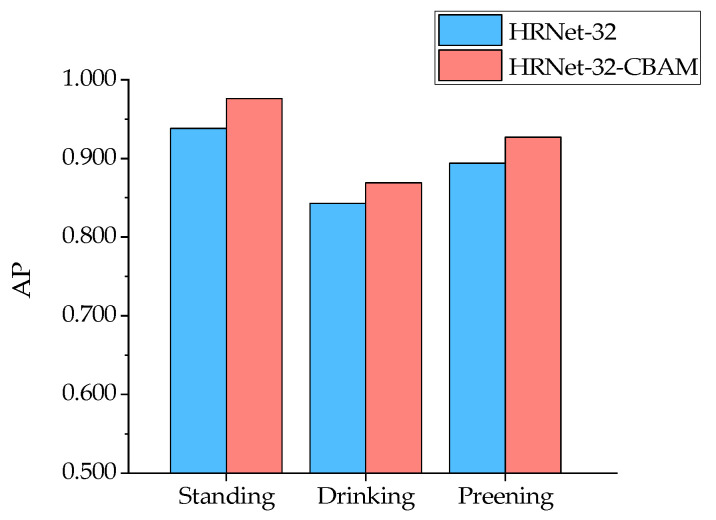
Comparison of AP for pose estimation of standing, drinking, and preening in HRNet-32 and HRNet-32-CBAM for ducks.

**Figure 16:**
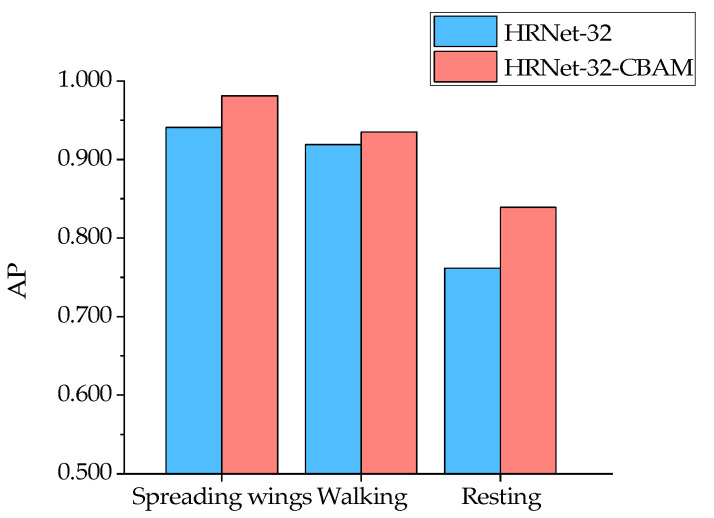
Comparison of AP for pose estimation of spreading wings, walking, and resting in HRNet-32 and HRNet-32-CBAM for ducks.

From the results in Figure 14, it can be seen that for the six types of duck poses, the HRNet-32-CBAM-based Jinling white duck pose estimation model can accurately detect and classify the eight types of keypoints in the duck body, and without any missed or false detections. At the same time, the keypoints are connected in the correct order to form the duck body without any incorrect expressions, indicating that the model can achieve accurate pose estimation for various behaviors of the Jinling white duck. In addition, to quantitatively analyze the performance distribution of the model for different poses, the experiment also obtained the AP values estimated by the model for six types of duck poses and further compared them with HRNet-32, as shown in Figure 15 and Figure 16.

The comparison results show that both HRNet-32-CBAM and HRNet-32 have high AP for standing and spreading wings and low AP for drinking and resting for the six poses of ducks. The reason may be that when the ducks are standing and spreading their wings, the keypoints of each part of the body can be fully visible in the image without any occlusion so that the corresponding features can be fully captured and learned by the model, which is conducive to the inference and localization of keypoint categories. In contrast, drinking and resting poses lead to various degrees of occlusion of keypoints of the beak, chest, left leg, right leg, left foot, and right foot of ducks, resulting in feature loss. Although the model can predict their approximate positions based on the symmetric and distribution patterns of the keypoints, there are still significant errors compared to manual annotations. Furthermore, the positive effect of the attention mechanism on the channel attention weight and spatial attention weight assigned to each resolution branch feature on the feature extraction and localization ability of the model also gives HRNet-32-CBAM advantages over HRNet-32 in the estimation of all six types of poses. Finally, for the AP values of the above six behaviors, HRNet-32-CBAM improves over HRNet-32 by 0.038, 0.026, 0.033, 0.04, 0.016, and 0.077, respectively.

### 3.3. Jinling White Duck Pose Estimation Results under Different Lighting Conditions

The image collection of the Jinling white duck was carried out under natural sunlight without specific requirements for front lighting and backlighting. Although the model proposed in this paper can achieve better accuracy in the above scenarios, the detection performance under low light conditions in the farm environment still needs to be determined. Therefore, it is necessary to test the generalization ability of the model.

**Figure 17:**
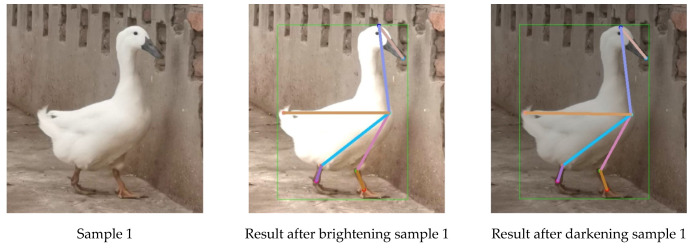

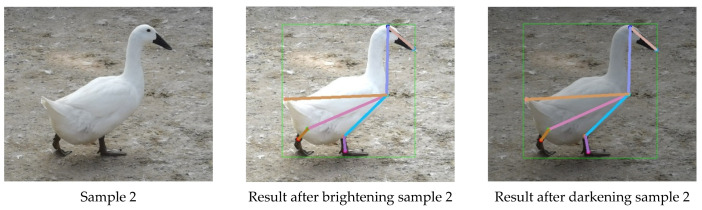
Pose estimation results of ducks under different lighting conditions.

The experiment used 100 randomly selected images of Jinling white duck to establish a test dataset to evaluate the model’s generalization ability, following the image brightness adjustment method in Section 2.4. The dataset contains a total of 200 “darkened” and “brightened” images. Subsequently, the generalization ability of the pose estimation model based on HRNet-32-CBAM was tested under “darkened” and “brightened” conditions, and the results are shown in Figure 17.

Based on the pose estimation results of samples 1 and 2 of Figure 17 under different lighting conditions, it can be seen that the model proposed based on HRNet-32-CBAM can accurately detect keypoints of different parts and categories of ducks, and the correlation of body structure is correct without an obvious error phenomenon. At the same time, even under occluded keypoints due to increased brightness in sample 2, accurate detection is still achieved. The above indicates that the change in light intensity does not significantly impact the model’s detection performance in this paper. The above indicates that changes in light intensity do not significantly impact the model’s detection performance in this paper. Finally, the model achieves an AP value of 0.856 for the generalization ability test dataset, indicating its applicability for Jinling white duck pose estimation under different lighting intensities and its strong generalization ability.

### 3.4. Pose Estimation Results for Different Breeding Ages

**Figure 18:**
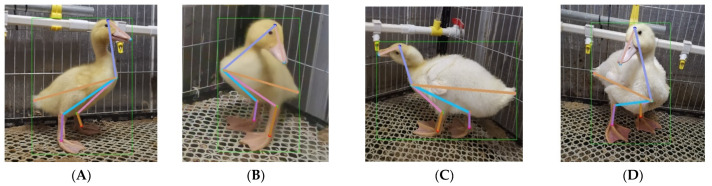
Pose estimation results of ducks at different breeding ages. (**A**) The sample 1 of 12 days breeding age duck. (**B**) The sample 2 of 12 days breeding age duck. (**C**) The sample1 of 24 days breeding age duck. (**D**) The sample 2 of 24 days breeding age duck.

Due to the significant differences in apparent color and body size between ducklings and adult ducks and considering that the duck pose estimation model is based on Jinling white ducks, this further increases the difficulty of the model in detecting postures of Cherry Valley ducklings. Therefore, based on HRNet-32-CBAM, the image dataset of ducks of different breeding ages was retrained to improve the robustness of the duck posture estimation model. Subsequently, the model was tested using 12- and 24-day-old ducklings with different breeding modes and breeds. Some results are shown in Figure 18.

As seen in Figure 18, as the age of breeding increases, the wing color of Cherry Valley ducks gradually lightens, and the size of various body parts significantly increases. This results in notable differences in duck images’ deep and superficial features. Meanwhile, the color of their beak is significantly different from that of the Jinling white duck, imposing higher requirements on the model’s detection performance. According to the results in Figure 18A–D, there are significant color differences between Figure 18B,D and distinct pose variations among Figure 18A,C,D. Despite these variations, the proposed model can still accurately detect eight keypoints of duck bodies of different reproductive ages and poses in a cage-reared mode without any missed or false detections. Furthermore, the correlation order of each keypoint is correct. Finally, regarding ducks of different breeding ages, the model’s AP value in this paper is 0.841, indicating its good detection performance for duck’s pose estimation across various breeding modes, ages, and breeds.

### 3.5. Comparative Analysis of Experimental Results

**Figure 19:**
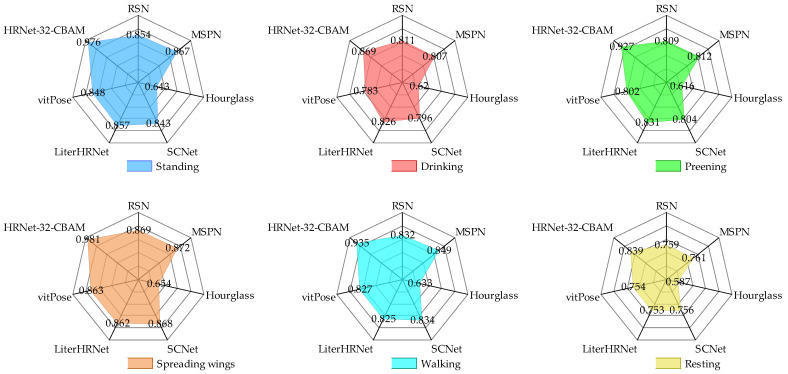
Comparison results of different pose estimation methods for multiple behavioral poses of ducks.

With the development of deep learning technology, an increasing variety of human and animal pose estimation methods have been developed, resulting in continuous improvements in detection accuracy. However, when dealing with different objects, the specific performance of each method often varies. To explore the advantages and disadvantages of the proposed method based on HRNet-32-CBAM compared to other commonly used methods, RSN [41], MSPN [42], hourglass [43], SCNet [44], LiterHRNet [45], and vitPose [46] were introduced to conduct comparative experiments on the pose estimation dataset. During the experiments, the computer platform, compilation environment, and training hyperparameters were kept consistent to ensure the environment’s consistency. The comparison results of AP values of the above six methods and HRNet-32-CBAM for the six poses of ducks standing, drinking, preening, spreading wings, walking, and resting are shown in Figure 19.

According to the results in Figure 19, for the six poses of ducks, RSN, MSPN, SCNet, LiterHRNet, and vitPose all achieved high AP values. They show the same pattern: the AP values for standing and spreading wings are higher than those for drinking, preening, walking, and resting. Moreover, the estimation values for the resting pose are relatively the lowest. The reason may be that the keypoints of standing and spreading wings are not obscured compared with the other four types of poses, thus facilitating the learning and localization of the features of each part of the duck. On the other hand, the key parts of the duck’s body have different degrees of mutual overlap when it rests, making it difficult to detect the keypoints.

**Figure 20:**
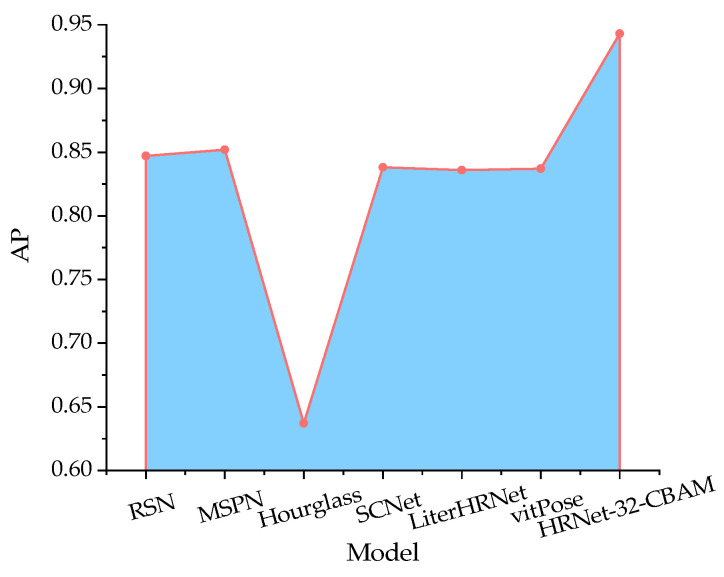
Comparison results of AP values for different methods.

Based on the distribution of radar map values, the highest AP values by the above five methods for standing, drinking, preening, spreading wings, walking, and resting poses were 0.857, 0.826, 0.831, 0.872, 0.849, and 0.761, respectively, which were 0.119, 0.043, 0.096, 0.109, 0.086, and 0.086 lower than those of the HRNet-32-CBAM. Therefore, except for the proposed model, MSPN and RSN had advantages over other methods for all types of duck pose estimation, but the overall differences were insignificant. In addition, the AP values obtained by the hourglass were 0.643, 0.620, 0.616, 0.654, 0.633, and 0.587, with an average decline of 0.212 relative to other methods and the worst detection performance. This indicates that the hourglass method does not apply to the six specific types of duck pose in the experimental setting of this paper. The AP values for the RSN, MSPN, hourglass, SCNet, LiterHRNet, and vitPose in the comparison tests are shown in Figure 20.

According to the results depicted in Figure 20, the AP values for RSN, MSPN, hourglass, SCNet, LiterHRNet, and vitPose were 0.847, 0.852, 0.637, 0.838, 0.836, and 0.837, respectively. The model proposed in this paper based on the HRNet-32-CBAM network achieved the highest AP values, which are 0.095, 0.091, 0.306, 0.105, 0.107, and 0.106 higher than those of the above six methods, respectively. This indicates that the proposed model outperforms others in the accuracy of estimating multiple types of poses for Jinling white ducks.

**Figure 21:**
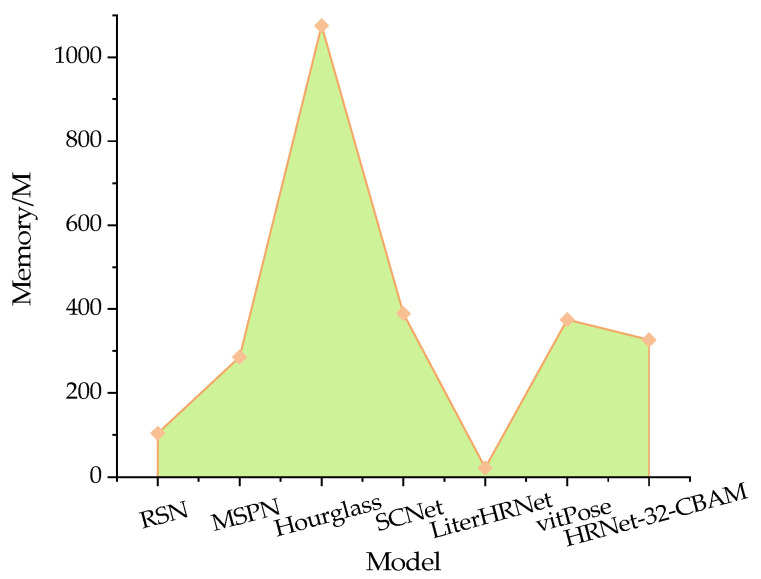
Comparison results of memory values for different methods.

The memory size of a model is directly related to its embeddability. To compare and analyze the embeddability of the proposed model, the results of the memory size of each method obtained experimentally are shown in Figure 21. In the figure, hourglass has the largest memory size, reaching 1075 M, while Liter-HRNet has the smallest memory size, at 22 M. The memory sizes of the other models range from 100 to 400 M. The proposed model in this paper has a memory size of 327 M, which is reduced by 69.58, 15.93, and 12.80% compared to hourglass, SCNet, and vitPose, respectively. However, it is 214.42, 14.33, and 1386.36% larger than those of RSN, MSPN, and LiterHRNet, respectively. This indicates that the proposed model has a certain level of embeddability.

### 3.6. Real-Time Testing Results

The Jinling white duck pose estimation model is required to have high accuracy and strong real-time performance. To further investigate the real-time performance of the proposed model in this paper and based on the principle of low resolution and low computational cost, some original image resolutions were resized to 256 × 256, 512 × 512, and 728 × 728 pixels at three different levels, and the number of image samples for each resolution was set to 24. Based on this, the average processing time of the model for individual images in different resolution image sample groups was recorded. This process was repeated three times, and the average values were calculated to obtain the final results of the real-time performance test. The results of the model’s real-time performance test are shown in Table 4.

From the results in Table 4, it can be seen that under the three pixel resolutions of 256 × 256, 512 × 512, and 728 × 728 pixels, the total time required for a quantitative duck image by the model proposed in this paper increases with increasing image resolution. Accordingly, the processing time for a single image increases. As the image resolution increases, the number of computational floating points of the model gradually increases, which increases the inference time for duck keypoint classification, localization, and body connection. Finally, the average processing time of a single image of the model in this paper is 0.265 s, indicating good real-time performance.

## 4. Discussion

### 4.1. The Impact of Different Network Widths on the Duck Pose Estimation Performance

The results demonstrated that for multi-pose estimation of the Jinling white duck, the proposed model based on HRNet-32 outperformed HRNet-48, as shown by the AP value increase of 0.004. The main difference between the HRNet-32 and HRNet-48 networks is the widths of the high-resolution subnetworks in the last three stages. As shown in Table 2, the width of the corresponding branch in HRNet-32 is smaller than that in HRNet-48. In general, larger feature maps are beneficial for the network to learn and correctly infer object features fully. However, in our experimental environment, the performance of the duck pose model obtained based on HRNet-48 is contrary to that. This indicates that for different experimental subjects and objectives, increasing the width of the HRNet network does not necessarily improve the model’s accuracy and may even result in no significant changes or a decrease in accuracy. Therefore, unquestioningly increasing the depth of neural networks to improve model performance is not advisable when facing different research needs.

### 4.2. The Influence of Different Duck Poses on Model Detection Accuracy

In this experiment, we detected a total of eight keypoints, including the head, beak, chest, left leg, right leg, left foot, right foot, and tail, for the six typical behaviors of ducks: standing, drinking, preening, spreading wings, walking, and resting. The duck pose estimation models proposed based on the original HRNet-32, and HRNet-32-CBAM showed high accuracy in detecting the standing and spreading wing poses. At the same time, they exhibited lower accuracy in estimating the drinking, preening, walking, and resting poses, as shown in Figure 15 and Figure 16. The comparative experiment results also demonstrated the same pattern, as shown in Figure 19. The above phenomenon may be because keypoints in various parts of the duck can be fully displayed in the image when standing and spreading its wings, while other keypoints in other poses may be obscured. Although the model can predict the approximate positions of the keypoints for drinking, preening, walking, and resting poses based on the symmetry and distribution patterns of the duck’s keypoint features, there are still deviations compared to the actual annotations.

### 4.3. The Impact of Introducing Attention Mechanisms on the Model Performance

We enhanced the contextual information by embedding the CBAM attention mechanism into the HRNet-32 network, turning the network focus on the most relevant regions to the duck’s keypoints to extract more discriminative features, thereby improving the keypoint detection accuracy of the model. The experimental results demonstrate that, in this experimental environment, utilizing the above method enhances the detection performance of the duck pose estimation model. Ultimately, the AP values of the model obtained based on HRNet-32-CBAM were improved by 0.038, 0.026, 0.033, 0.040, 0.016, and 0.077 for the six typical behaviors of ducks standing, drinking, preening, spreading wings, walking, and lying, respectively, compared with HRNet-32. This finding can provide reference ideas for experiments with low model detection accuracy, limited sample size, and the need for further performance improvement.

### 4.4. The Proposed Method’s Limitations and Future Approaches for Resolution

The experimental results show that the proposed method can achieve multi-class pose estimation of the Jinling white duck and demonstrates excellent generalization ability. Furthermore, it still shows high accuracy in pose recognition for ducks of different breeding ages, breeding modes, and breeds, outperforming other commonly used methods in comprehensive performance and real-time capability. However, there are still some limitations of this method:

(1) The Jinling white duck pose estimation method proposed in this paper is based on implementing a single duck. The single duck breeding mode is commonly used in the breeder duck farming process, which differs from the large-scale group-reared and cage-reared modes. The research findings of this paper are the premise and foundation for the subsequent use of computer vision methods to analyze duck behavior in complex scenes.

(2) The duck pose estimation dataset labels are manually annotated, which is labor-intensive and time-consuming. In the future, we will focus on semi-supervised research to achieve higher model performance with fewer samples and reduced manual costs.

(3) The duck category is too limited. In the future, there should be an increase in the diversity of applicable duck breeds to enhance the robustness of the model and expand its application scenarios.

## 5. Conclusions

This paper proposed a Jinling white duck pose estimation method based on HRNet-32-CBAM. The results show that this method can accurately detect multiple behaviors, pose keypoints of ducks, and form correct body expressions. For the Cherry Valley ducks at the ages of 12 and 24 days, as well as the Jinling white duck pose estimation under varying lighting conditions, the proposed method achieved AP values of 0.841 and 0.856, respectively, with a processing time for a single image of 0.256 s. These results prove the proposed method’s high adaptability, generalization, and real-time detection capability. The model’s detection accuracy can be improved by embedding the attention mechanism CBAM. The proposed model based on HRNet-32-CBAM achieved an AP improvement of 0.048 compared to HRNet-32. When the key parts of the duck are under different occlusion situations, the corresponding AP values are relatively lower, especially in the duck’s resting pose cases. The proposed method establishes a technical foundation for future pose estimation and behavior analysis of group-reared and cage-reared ducks. Furthermore, it can also provide a technical reference for the intelligent farming of other poultry animals.

## Figures and Tables

**Table 1:** Description and definition of keypoints of a Jinling white duck.

Labels	Custom Keypoints Names	Keypoints Description	Corresponding Color (RGB)
T	Head	Duck crown feather region	(255,0,0)
H	Beak	Bill knob area of the duck’s beak	(255,165,0)
X	Chest	Duck chest feather region	(255,255,0)
LG	Left leg	Junction of the left leg and plumage of the duck	(0,255,0)
RG	Right leg	Junction of the right leg and plumage of the duck	(0,125,255)
LJ	Left foot	Junction of the left leg and left foot in the duck	(0,0,255)
RJ	Right foot	Junction of the right leg and right foot in the duck	(139,0,255)
W	Tail	Duck tail feather region	(192,0,0)

**Table 2:** Description of duck pose by professionals in duck farming.

Pose	Pose Judgment Standard
Standing	Body is vertical, with feet flat on the ground. The neck is usually upright or curved upward, and wings are pressed against the body. The legs are upright.
Drinking	The body approaches the water surface, bringing the bill near or immersed in the water. The wings are pressed tightly against the body, and the legs are slightly bent.
Preening	The beak is positioned near the chest feather area or wings, with the neck curved, and the legs slightly bent or upright, while the wings are slightly extended.
Spreading wings	In a standing pose, the wings are fully extended, and the necks are raised.
Walking	Wings are slightly extended or pressed against the body, the neck is raised or curved in an S-shape, one foot is off the ground, or in contact at a certain angle
Resting	The neck is curved, or the head is placed within the wings, while the legs and feet are typically folded beneath the body.

**Table 3:** Width of the Stage 2–Stage 4 in HRNet-32 and HRNet-48.

Number of Stage	HRNet-32	HRNet-48
Stage 2	64	96
Stage 3	128	192
Stage 4	256	384

**Table 4:** Real-time performance testing results of duck pose estimation models based on HRNet-32-CBAM.

Image Resolution	Average Total Detection Time Consuming/s	Average Processing Time of Single Image/s
256 × 256	5.712	0.238
512 × 512	6.433	0.268
728 × 728	6.925	0.289
Mean	6.356	0.265

## Data Availability

Data are contained within the article.
